# A Novel Chimeric Oncolytic Virus Mediates a Multifaceted Cellular Immune Response in a Syngeneic B16 Melanoma Model

**DOI:** 10.3390/cancers16193405

**Published:** 2024-10-06

**Authors:** Sonja Glauß, Victoria Neumeyer, Lorenz Hanesch, Janina Marek, Nina Hartmann, Gabriela M. Wiedemann, Jennifer Altomonte

**Affiliations:** Department of Internal Medicine II, Rechts der Isar Hospital, Technical University of Munich, 81675 Munich, Germany; sonja.glauss@tum.de (S.G.); victoria.neumeyer@tum.de (V.N.); lorenz.hanesch@tum.de (L.H.); janina.marek@tum.de (J.M.); nina.hartmann@campus.lmu.de (N.H.); gabriela.wiedemann@tum.de (G.M.W.)

**Keywords:** oncolytic virotherapy, cancer immunotherapy, flow cytometry

## Abstract

**Simple Summary:**

Cancer immunotherapy has evolved rapidly in the last decade. A newly emerging immunotherapeutic approach utilizes oncolytic viruses to enhance the ability of the body’s own immune system to recognize and clear tumor cells. We have recently developed a novel chimeric oncolytic virus and demonstrated its anti-tumoral effects in various preclinical mouse models. To gain mechanistic insights into the immune response to this form of therapy at the cellular level and to identify targets to potentially further improve the therapy, we analyzed the immune cell signature in the tumors, blood, and lymphoid tissue in a syngeneic melanoma mouse model by flow cytometry. Both local and systemic immune responses were observed. Furthermore, selective depletion of cytotoxic T cells indicated that these cells are the main players in mediating the therapeutic response to this novel virotherapy.

**Abstract:**

Background/Objectives: Oncolytic virotherapy is a promising approach in cancer immunotherapy. We have previously described a recombinant hybrid oncolytic virus (OV), VSV-NDV, which has a favorable safety profile and therapeutic immunogenicity, leading to direct oncolysis, abscopal effects, and prolonged survival in syngeneic in vivo tumor models. While OVs are known to mediate systemic anti-tumor immune responses, the detailed characterization of local and systemic immune responses to fusogenic oncolytic virotherapy remains unexplored. Methods and Results: We analyzed immune cell compartments in the spleen, blood, tumor-draining lymph nodes (TDLNs), and tumors over the course of VSV-NDV therapy in a bilateral syngeneic melanoma mouse model. Our results revealed significant local infiltration and activation of T lymphocytes in tumors and globally in the blood and spleen. Notably, in vivo CD8^+^ T cell depletion led to complete abrogation of the tumor response, highlighting the crucial role of T cells in promoting the therapeutic effects of oncolytic VSV-NDV. In vitro co-culture experiments enabled the interrogation of human immune cell responses to VSV-NDV-mediated oncolysis. Human peripheral blood mononuclear cells (PBMCs) were efficiently stimulated by exposure to VSV-NDV-infected cancer cells, which recapitulates the in vivo murine findings. Conclusions: Taken together, these data characterize a broad anti-tumor immune cell response to oncolytic VSV-NDV therapy and suggest that CD8^+^ T cells play a decisive role in therapeutic outcome, which supports the further development of this chimeric vector as a multimechanistic immunotherapy for solid cancers.

## 1. Introduction

Over the last decade, the field of oncolytic virus (OV) therapy has experienced remarkable progress, highlighted by significant clinical advancement and expanding therapeutic applications. The approval of the first oncolytic virus, a recombinant herpes simplex virus (T-VEC), by the United States Food and Drug Administration (FDA) and the European Medicines Agency (EMA) for the treatment of metastatic melanoma, nearly ten years ago, marked a pivotal advancement [1]. However, the marketing progress since then has been slow, and the next OV therapy approval occurred in 2021, this time for malignant glioma, and sanctioned solely in Japan [2]. This trajectory, although slower than expected, is nevertheless underscored by the exponential growth in clinical studies and has resulted in a growing accumulation of clinical data that validate the immunotherapeutic potential of oncolytic virus therapies, although adenoviruses have been by far the most commonly used OV vector, and melanoma, glioma, and gastrointestinal cancer are among the most targeted types of cancer [3]. These studies have generally demonstrated that OVs are well tolerated, even in an immune-suppressed setting, with mostly mild and acceptable toxicities having been reported [4].

Despite these important advancements, most OV candidates are still in the early stages of preclinical or clinical (phase I or II) development [5]. Nevertheless, it is becoming increasingly evident that OV approaches hold immense promise as cancer therapies, owing to their potent therapeutic effects and favorable safety profile, primarily attributed to their tumor-selective replication mechanisms. A critical aspect responsible for the tumor selectivity of RNA-based oncolytic viruses is the acquired deficiencies in antiviral type I interferon signaling (IFN) pathways within tumor cells, contributing to evasion from immune surveillance, while also facilitating viral propagation [6]. Conversely, the intact antiviral defense mechanisms in healthy cells serve to restrain viral replication by inducing an orchestrated transcriptional response mediated by IFN-signaling elements [7,8]. In addition their direct oncolytic effects, it has more recently been shown that OVs can additionally modulate the tumor microenvironment, transforming it from an immunologically “cold” to “hot” milieu [9]. This shift is attributed to innate immune responses against the virus and the phenomenon of immunogenic cell death (ICD), characterized by the release of danger-associated molecular patterns (DAMPs) and tumor-associated antigens (TAAs) upon tumor cell lysis [10]. The recognition of DAMPs by innate immune cells triggers an intricate cascade of events, culminating in the cross-presentation of TAAs by antigen-presenting cells (APCs) to cytotoxic CD8^+^ T cells, thereby eliciting a robust adaptive immune response to target local and distant tumors and micro-metastases [11]. In contrast to the TCR-dependent activation of T cells, a TCR-independent and cytokine-dependent mechanism of T cell activation as a response to viral infection has been described as bystander activation [12]. Type I interferon leads to the expansion of CD8^+^ T cells; however, the precise mechanism of this activation pathway has not yet been fully elucidated [13].

While the central role of immune stimulation in oncolytic virotherapy has been described, a comprehensive in vivo characterization of the immune response after OV treatment, encompassing local responses in treated tumors, as well as systemic responses in spleen, draining lymph nodes, blood, and distant tumors is imperative in order to better understand the involvement of different immune cell types and their respective localizations. This would allow a more detailed mechanism of action and characterization of the immune-modulatory aspect of the therapy and the development of rationally designed next-generation vectors and combination therapies to optimize these effects.

We have previously reported on a novel chimeric OV construct, comprising the vesicular stomatitis virus (VSV) backbone and the Newcastle disease virus (NDV) envelope proteins [14]. This construct demonstrated superior safety and efficacy compared to its parental viruses, offering enhanced anti-tumor immune stimulation through its ability to mediate highly immunogenic cell death via syncytia formation [14,15]. Moreover, it was shown to synergize with anti-CTLA-4 treatment and adoptive cell therapy (ACT), resulting in improved tumor responses and prolonged survival [15,16].

In this study, we conducted an in-depth characterization of the cellular immune response to oncolytic VSV-NDV using the murine B16OVA system, employing chicken ovalbumin as an immunogenic model antigen in order to distinguish between tumor-associated antigen-specific, virus-specific, and overall T cell responses. The analysis of distant and directly treated tumors revealed a significant increase in activated CD4^+^ and CD8^+^ T cells and tumor-specific CD8^+^ T cells upon intratumoral VSV-NDV treatment, demonstrating the transition from a “cold” to a “hot” tumor microenvironment. In the local TDLNs, we observed an early increase in the influx and activation of all examined immune cell types. Importantly, VSV-NDV treatment also induced a systemic enhancement of T cell responses in peripheral blood and in the spleen, and CD8^+^ T cell depletion completely reversed VSV-NDV-mediated anti-tumor effects, elucidating a critical mechanism of action of this form of therapy. These results unveil a multifaceted systemic immune response as a crucial component of oncolytic VSV-NDV therapy, thereby supporting this fusogenic virus as a promising platform for cancer immunotherapy.

## 2. Materials and Methods

### 2.1. Tumor Cell Lines and Virus

B16OVA and HepG2 cells (generous gifts from Simon Heidegger and Ulrike Protzer, respectively, from Klinikum rechts der Isar, Munich, Germany) were cultured in complete Dulbecco’s modified Eagle’s medium (DMEM) supplemented with 10% heat-inactivated fetal calf serum (FCS), minimum essential medium (MEM) non-essential amino acids, and sodium pyruvate. VSV-NDV was generated as described previously [14]. Virus stocks used for the experiments were produced in adherent AGE1.CR.pIX cells (ProBioGen, Berlin, Germany) and purified by ultracentrifugation over a sucrose gradient. Purified virus was resuspended in PBS after an additional ultracentrifugation step, and stocks were stored at −80 °C until use. Viral titers were determined by TCID50 assay in AGE1.CR.pIX cells.

### 2.2. Mouse Studies

All animal studies were approved by the institute’s commission for preclinical animal research and the regional government commission for animal protection (Regierung von Oberbayern, Munich, Germany). Female C57Bl/6J mice aged 6–8 weeks (Janvier Labs, France) were maintained under specific pathogen-free conditions. A subcutaneous bilateral tumor model was used for survival and mechanistic endpoint analysis. Mice were shaved and then injected subcutaneously with B16OVA into the right (2.4 × 10^5^ cells) and left (1.2 × 10^5^ cells) flanks in a volume of 50 µL of PBS. One week later (day 0), when tumors were visible (tumor volume approximately 20–50 mm^3^), mice were treated by intratumoral injection of 50 µL of VSV-NDV (1 × 10^7^ TCID50) or PBS into the tumor located on the right lateral flank (injected tumor), repeated on days 3 and day 6. For survival experiments, mice were monitored daily and euthanized when the maximum tumor diameter exceeded 15 mm or tumor rupture occurred, according to the requirements of local regulatory agencies. On days 2 and 10 after the first treatment, additional cohorts of mice were euthanized, tissue samples were collected, and single-cell suspensions were generated. Blood was collected in EDTA-microvettes (Sarstedt), and red blood cell (RBC) lysis was performed with RBC buffer (BioLegend, San Diego, CA, USA). Single-cell suspensions of dispersed tissue were used for flow cytometry. For cell depletion experiments, 100 µg of anti-CD8 antibody or IgG isotype control (both Bio X Cell) were initially given one day pre-treatment start. Complete depletion was confirmed on the first day of virus treatment by flow cytometry, and a survival experiment was conducted as described above.

### 2.3. RT-qPCR

Tumor tissue was collected and homogenized using a Precellys lysing kit (Bertin Technologies, Montigny-le-Bretonneux, France). RNA was isolated using a Maxwell RSC SimplyRNA tissue kit (Promega, Madison, WI, USA) and the Maxwell RSC instrument (Promega). GoTaq 1-step RT-qPCR Kit (Promega) and a 7500 Real Time PCR system (Applied Biosystems, Foster City, CA, USA) were used to assess transcript abundance. Samples were quantified in duplicates using 50 ng of template RNA and the following cycling conditions: reverse transcription (RT) at 50 °C for 15 min; reverse transcriptase inactivation/hot-start polymerase activation at 95 °C for 10 min, followed by 40 cycles of 95 °C for 15 s (denaturation), 60 °C (IFN-γ, IL-15, T-bet, IFN-β, and GAPDH) or 53 °C (CXCL10) for 30 s (annealing), and 72 °C for 30 s (extension). GAPDH was used as a housekeeping gene for normalization, and the comparative ΔΔC_T_ method was used to calculate target gene expression. Primer sequences for amplification of the respective genes are shown in Table 1.

### 2.4. In Vitro Co-Culture

HepG2 cells were infected with VSV-NDV at an MOI of 0.1 or 1, in RPMI 1640 Medium GlutaMAX-I (Invitrogen, Waltham, MA, USA), supplemented with 10% heat-inactivated FCS, MEM non-essential amino acids, sodium pyruvate, and β-mercaptoethanol (50 mM) and incubated for 16 h, before adding PBMCs isolated from human blood collected from healthy volunteers (ethics approval under file #318/19 S-SR, granted by the institutional review board of Klinikum rechts der Isar), followed by a 24 h incubation before cells were harvested and stained with flow cytometry antibodies.

### 2.5. Flow Cytometry

The following antibodies (BioLegend, San Diego, CA, USA) were used for staining for flow cytometry: anti-CD3e (145-2C11), anti-CD4 (GK1.5), anti-CD11b (M1/70), anti-CD11c (N418), anti-CD19c (1D3/CD19), anti-CD44 (IM7), anti-CD69 (H1.2F3), anti-CD86 (GL-1), anti-CD122 (5H4), anti-F4/80 (BM8), anti-H-2Db (KH95), anti-I-A/I-E (M5/114.15.2), anti-IFNγ (XMG1.1), anti-NK-1.1 (PK136), anti-NKG2D(CX5), and anti-PD-1 (29F.1A12). Additionally, the following antibodies (Miltenyi Biotech, Bergisch Gladbach, Germany) were used: anti-CD3 (REA641), anti-CD8 (REA601), anti-CD49a (REA493), anti-CD62L (REA828), and anti-CD69 (H1.2F3), as well as Viobility™ 405/520 Fixable Dye. From Sony, anti-CD49b (DX5) was used and from Invitrogen, anti-CD103 (2E7) was used. The H-2kb OVA Tetramer-SIINFEKL (MBL International, Woburn, MA, USA) was used. We thank the NIH Tetramer Core Facility (contract number 75N93020D00005) for providing I-Ab OVA Tetramer-AAHAEINEA, H-2kb VSV Tetramer-RGYVYQGL, H-2kb OVA Tetramer-SIINFEKL, mouse CD1d Tetramer loaded with the α-GalCer analog PBS57. For human in vitro experiments, the following antibodies (BioLegend) were used: anti-CD3 (UCHT1), anti-CD4 (RPA-T4), anti-CD56 (5.1H11), and anti-CD69 (FN50). Also, anti-CD8 (BW135/80) from miltenyi and anti-IFNγ (B27) from BD Pharmingen were used. For mouse in vitro experiments, anti-IFNγ (XMG1.2) from BioLegend was additionally used.

For experiments including the AAHAEINEA(OVA)-specific tetramer, isolated cells were incubated for 2 h at 37 °C, whereas for experiments including MHC-I tetramers, isolated cells were incubated for 20 min at room temperature with SIINFEKL(OVA)-/(VSV-NP)-specific tetramers. Extracellular staining was performed for 30 min at 4 °C, washed with PBS, optionally fixed and intracellularly stained with BD Cytofix/Cytoperm ™ Fixation/Permeabilization Kit (BD Biosciences, Franklin Lakes, NJ, USA) according to the manufacturer’s protocol.

CountBright™ Absolute Counting Beads (Invitrogen™) were used for the calculation of the event count. Flow cytometric measurements were performed using the CytoFLEX S platform (Beckman Coulter Genomics, Brea, CA, USA). The compensation was performed based on staining results from UltraComp beads (Thermo Fisher Scientific, Waltham, MA, USA). All flow cytometry results were analyzed using Flow Jo v10.8 (Ashland, OR, USA) software.

### 2.6. Statistical Analysis

All data were plotted and analyzed using GraphPad Prism 10.0 (GraphPad, San Diego, CA, USA). Individual data points were compared for statistical significance using an unpaired Student’s *t* test or ordinary one-way ANOVA for multiple groups, and *p* values of less than 0.05 were considered to be statistically significant (* *p* < 0.05, ** *p* < 0.01, *** *p* < 0.001, **** *p* < 0.0001). Survival data were plotted as Kaplan–Meier curves, and statistical significance was calculated by log rank test.

## 3. Results

### 3.1. VSV-NDV Reduces the Tumor Mass and Induces the Expression of Inflammatory Markers in a Syngeneic B16 Melanoma Model

**Figure 1:**
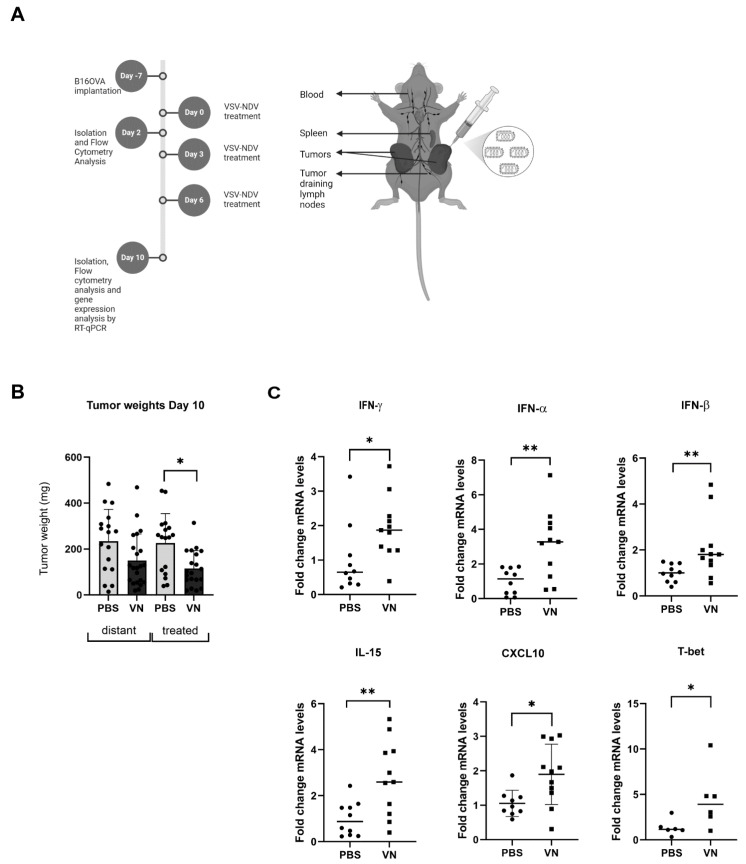
VSV-NDV reduces the tumor mass and mediates the expression of cytokines in murine melanoma. (**A**) The experimental set-up of the mechanistic endpoint experiment. C57BL6/J mice were implanted with B16OVA cells subcutaneously on contralateral flanks. Seven days later, the mice were randomly distributed into treatment groups (N = 6–8) and injected intratumorally with VSV-NDV (VN) at a dose of 1 × 10^7^ TCID50 or PBS in an equal volume of 50 µL on day 0, 3, 6. Tumors were collected on days 2 and 10 post-treatment start. Created in BioRender. Altomonte, J. (2024) https://BioRender.com/p46u790. (**B**) Tumor weight was measured on day 10. (**C**) Quantitative real-time RT-PCR was performed to measure intratumoral mRNA expression on day 2 (IL-15, IFN-γ, IFN-α, IFN-β, and CXCL10) or day 10 (T-bet). Relative gene expression was quantified by normalization to GAPDH using the 2^−ΔΔCt^ method. Data points indicate values of individual replicates (and group means are presented as bars); * indicates *p* ˂ 0.05, and ** indicates *p* ˂ 0.01).

Based on our previous findings, which demonstrate that VSV-NDV treatment leads to tumor control and survival prolongation in the syngeneic B16 melanoma mouse model, we sought to investigate changes in intratumoral gene expression levels of inflammatory factors in response to OV treatment as a first glimpse into the mechanism. To this end, we performed the tumor implantation and treatment schedule as previously described, and euthanized cohorts of mice on day 2 and 10 post-treatment (Figure 1A). First, we validated the therapeutic effect on tumor size by comparing tumor weights, which demonstrated a reduction in tumor mass in response to VSV-NDV treatment in comparison to PBS treatment on day 10 post-treatment, irrespective of whether the tumor was directly injected or on the contralateral flank, but only reached statistical significance for the directly treated tumor (Figure 1B). Quantitative RT-PCR (RT-qPCR) analysis revealed higher mRNA levels of interleukin 15 (IL-15), Interferon-γ (IFN-γ), IFN-α, and IFN-β, as well as the migration marker, CXCL10, in the tumors directly treated by VSV-NDV compared to PBS-treated tumors on day 2 post-treatment start, while mRNA levels of T-bet were significantly increased on day 10 post-treatment start (Figure 1C). This suggests that early (innate) immune signaling to VSV-NDV treatment is triggered within the tumor.

### 3.2. VSV-NDV Treatment Leads to an Increase in Activated Tumor-Specific CD8^+^ T Cells and Memory and Effector T Cells

**Figure 2:**
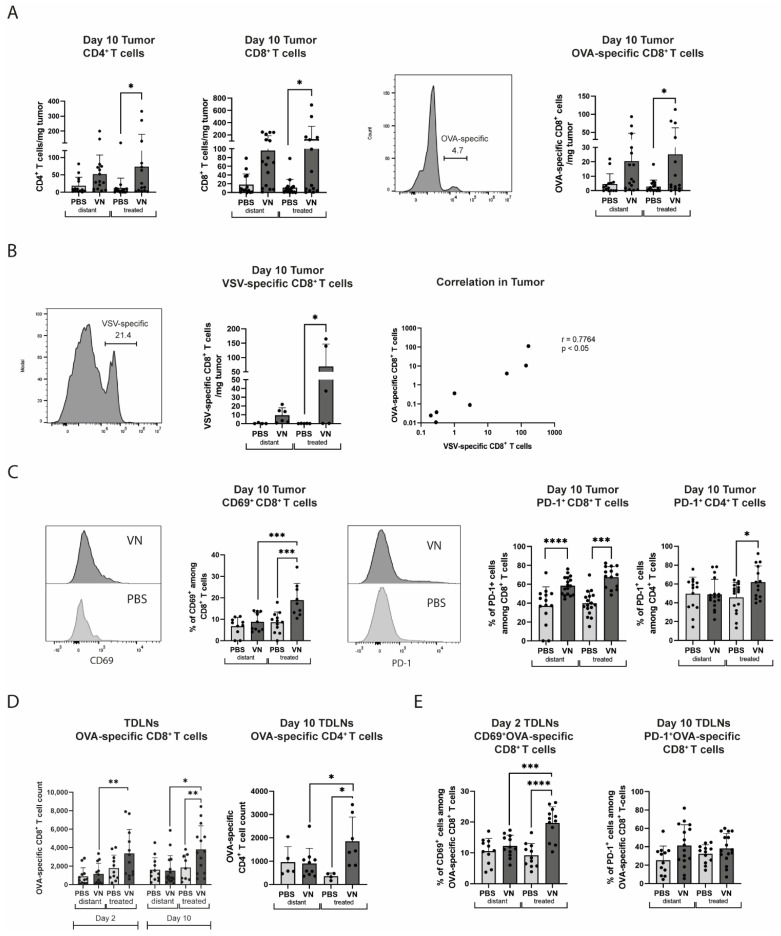
VSV-NDV treatment leads to T cell activation in tumors and lymph nodes. Bilateral B16OVA-bearing mice were treated intratumorally with VSV-NDV or PBS and analyzed by flow cytometry. (**A**) CD8^+^, CD4^+^, and OVA-TCR^+^CD8^+^ T cells in the tumor on day 10. (**B**) VSV-TCR^+^CD8^+^ T cells and the correlation of VSV^+^TCR and OVA^+^TCR in the tumor on day 10. (**C**) CD69 expression on CD8^+^ T cells on day 10 in the tumor and PD-1 expression on CD4^+^ and CD8^+^ T cells on day 10 in the tumor. (**D**) OVA-TCR^+^CD8^+^ or OVA-TCR^+^CD4^+^ T cells on day 2 and day 10. (**E**) CD69 expression on day 2 and PD-1 expression on day 10 of OVA-TCR^+^CD8^+^ T cells. N = 5–15 mice; data points indicate values of individual replicates (and group means + SD are presented as bars); * indicates *p* ˂ 0.05, ** *p* ˂ 0.01, *** *p* ˂ 0.001, **** *p* ˂ 0.0001.

We next sought to investigate changes in immune cell signatures over the course of oncolytic VSV-NDV treatment. An analysis of CD4^+^, CD8^+^, and OVA-specific CD8^+^ T cells in the tumor on day 10, but not on day 2 post-treatment start, revealed an increase in numbers in both directly injected and distant tumors in mice treated with VSV-NDV that reached statistical significance for the treated tumor (Figure 2A). Additionally, a notable, statistically significant increase in VSV-specific CD8^+^ T cells in virus-treated mice was observed in the treated tumor (Figure 2B). Interestingly, we observed a positive correlation between the number of VSV-specific and OVA-specific CD8^+^ T cells in the treated tumors of individual mice. Therefore, although VSV-NDV seems to elicit a substantial antiviral T cell response, this effect does not seem to detract from the induction of a potent CD8^+^ T cell response directed against the tumor. Furthermore, significant increases in CD69 expression levels in the treated tumor and PD-1 expression levels in both tumors on CD8^+^ T cells were detected, indicating activation. PD-1 expression, but not CD69 expression, was significantly enhanced on CD4^+^ T cells in the treated tumor (Figure 2C). The investigation of the TDLNs revealed a significant increase in OVA-specific CD8^+^ T cells on the treated side on days 2 and 10 in VSV-NDV treated mice, while a significant enrichment of OVA-specific CD4^+^ T cells was only observed at the later timepoint (Figure 2D). The OVA-specific CD8^+^ T cells also showed a significantly higher expression level of CD69 on the treated side on day 2, whereas a trend of higher PD-1 expression was seen in the TDLN on both sides of VSV-NDV treated mice (Figure 2E).

**Figure 3:**
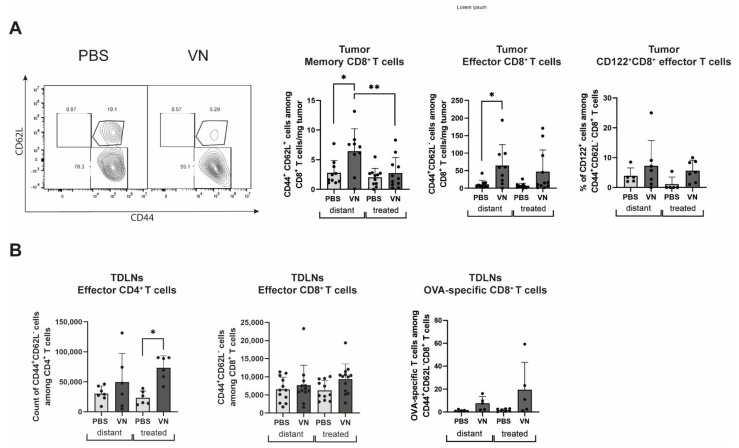
Intratumoral VSV-NDV treatment modulates CD44/CD62L expression patterns on T cells in B16 melanoma lesions and tumor-draining lymph nodes (TDLN). Bilateral B16OVA lesions and TDLNs were isolated from female C57Bl/6 mice treated with rVSV-NDV or PBS on day 10 post-treatment and analyzed by flow cytometry. (**A**) CD44/CD62L expression on CD8^+^ T cells and CD122^+^CD44^+^CD62L^+^CD8^+^ T cells on day 10 in the tumor. (**B**) CD44^+^CD62L^−^CD4^+^, CD44^+^CD62L^−^CD8^+^, and OVA-specific CD44^+^CD62L^+^CD8^+^ T cells on day 10 in the TDLNs. N = 5–15; data points indicate values of individual replicates (and group means + SD are presented as bars); * indicates *p* ˂ 0.05, ** indicates *p* ˂ 0.01.

We further investigated the memory and effector CD8^+^ T cell compartments by analyzing expression patterns of CD62L and CD44 (Figure 3A). A significant increase in memory CD8^+^ T cells was found in the distant tumor in comparison to the treated tumors of the VSV-NDV-treated mice, while an increase in effector CD8^+^ T cells was observed in both tumors of the virus-treated mice compared to control mice. The effector population also displayed a higher expression of CD122, the beta subunit of the interleukin-2 receptor, in both tumors, although the differences were not statistically significant (Figure 3A). In the TDLNs of the virus-treated tumors, effector CD4^+^ T cells were increased compared to all other controls (Figure 3B), while for effector CD8^+^ T cells a trend towards an increase was observed. Furthermore, within the effector CD8^+^ T cell subset in the distant tumors, there was also an increase in OVA-specific cells in response to virus treatment compared to PBS (Figure 3B).

### 3.3. Intratumoral VSV-NDV Treatment Leads to an Enhanced Antigen-Specific T Cell Response in Peripheral Blood and Spleen

**Figure 4:**
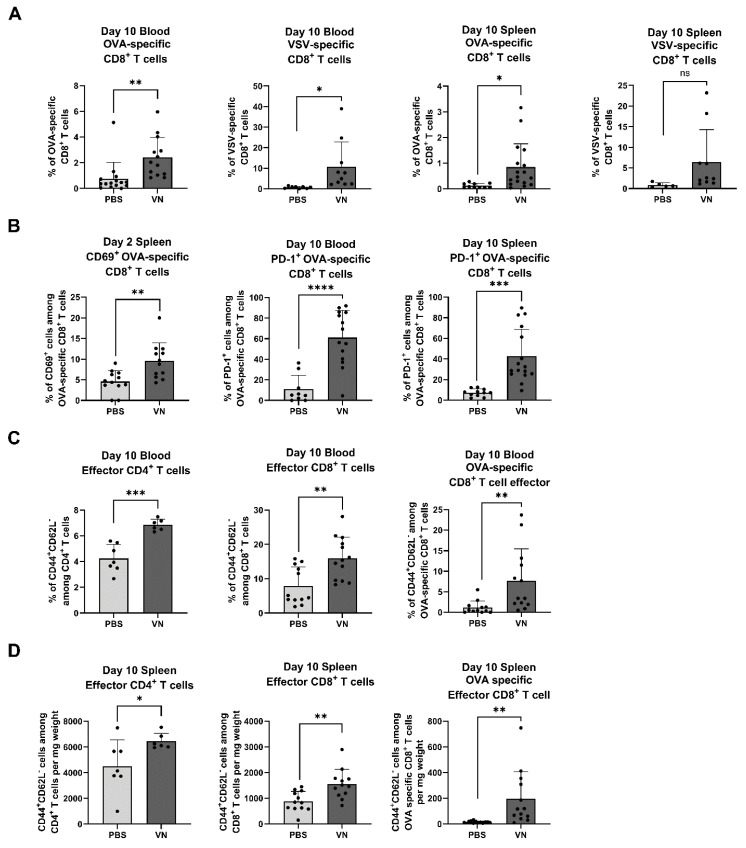
Local VSV-NDV treatment mediates systemic immune-stimulatory effects. C57BL6/J mice were implanted with B16OVA cells subcutaneously on contralateral flanks and, 7 days later, injected intratumorally with VSV-NDV (VN) at a dose of 1 × 10^7^ TCID50 or PBS and analyzed by flow cytometry for (**A**) OVA-TCR^+^CD8^+^ and VSV-TCR^+^CD8^+^ T cells on day 10, (**B**) CD69 expression on OVA-TCR^+^CD8^+^ T cells in the spleen on day 2 and PD-1^+^OVA^+^CD8^+^ T cells in the blood and spleen on day 10, (**C**) CD44^+^CD62L^−^ expression on CD4^+^, CD8^+^, and OVA-TCR^+^CD8^+^ T cells on day 10 in blood, or (**D**) in the spleen. Data points indicate values of individual replicates (and group means ± SD are presented as bars); * indicates *p* ˂ 0.05, ** *p* ˂ 0.01, *** *p* ˂ 0.001, **** *p* ˂ 0.0001.

As we had observed evidence of abscopal effects, we were prompted to further investigate the systemic immune response to local VSV-NDV therapy. An analysis of blood and spleen on day 10 post-treatment start revealed an increase in the percentage of OVA- and VSV-specific CD8^+^ T cells in mice treated with VSV-NDV compared to PBS (Figure 4A). Additionally, CD69 was significantly upregulated on OVA-specific CD8^+^ T cells as early as day 2 in the spleen, while PD-1 was upregulated on these cells and on the VSV-specific CD8^+^ T cells in both blood and spleen on day 10 after VSV-NDV treatment (Figure 4B). The analysis of the effector cell markers revealed significant increases in effector CD4^+^, CD8^+^, and OVA-specific CD8^+^ T cells in the peripheral blood and spleen of VSV-NDV-treated mice (Figure 4C,D).

### 3.4. VSV-NDV Elicits an Innate Immune Response in the TDLNs, Spleen, and Tumors

**Figure 5:**
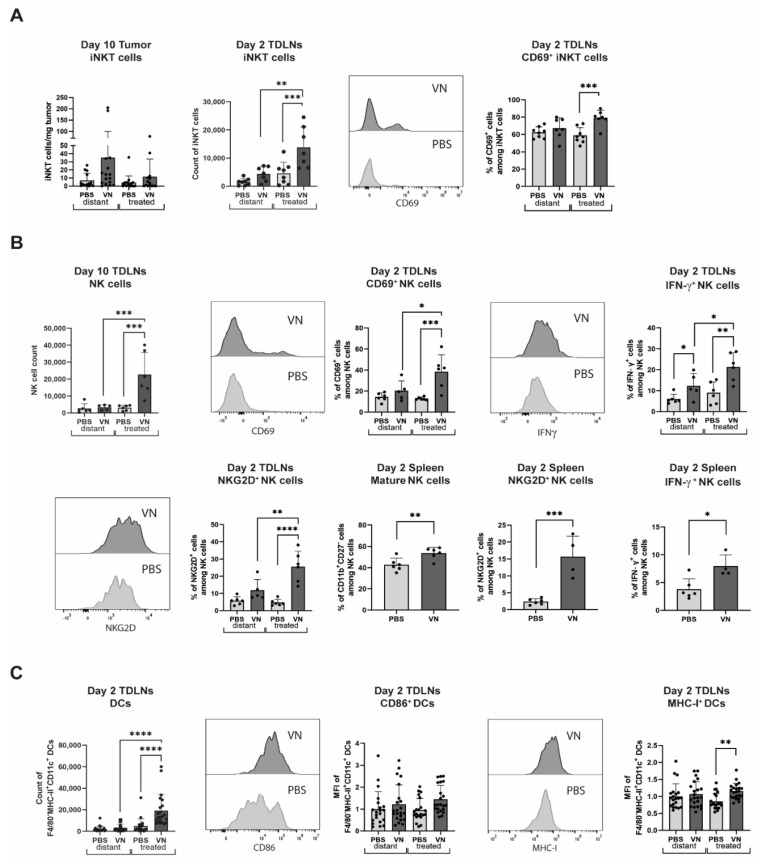
The intratumoral VSV-NDV treatment of B16 melanoma induces the enrichment and activation of innate immune cells. C57BL6/J mice were implanted with B16OVA cells subcutaneously on contralateral flanks. After seven days, the mice (N = 6–8) were injected intratumorally with VSV-NDV (VN) at a dose of 1 × 10^7^ TCID50 or PBS in 50 µL on day 0, 3, 6. TDLNs or tumors were analyzed by flow cytometry for (**A**) for iNKT cell number and CD69 expression, (**B**) NK cell number, CD69 expression, IFN-γ expression, and NKG2D expression in the TDLNs and mature NK cells, NKG2D^+^ NK cells, and IFN-γ in the spleen, or (**C**) for DC number, CD86 expression, and MHC-I expression in TDLNs. Data points indicate values of individual replicates (and group means ± SD are presented as bars); * indicates *p* ˂ 0.05, ** *p* ˂ 0.01, *** *p* ˂ 0.001, **** *p* ˂ 0.0001.

Since the innate arm of the immune system is known to be quickly activated in response to virus infection, we next investigated the innate cellular response to oncolytic rVSV-NDV treatment. While flow the cytometry analysis of the tumors did not reveal differences in numbers of DCs or NK cells, an increase in invariant natural killer T (iNKT) cells after VSV-NDV treatment was detected on day 10 post-treatment start that was strongest for the distant tumor, although not statistically significant (Figure 5A). CD69 expression was similar between the groups. Additionally, a significant increase in iNKT cells in the TDLNs of the treated sides was revealed when comparing VSV-NDV-treated mice to those receiving PBS on day 2 post-treatment. Notably, this population also demonstrated a significantly higher expression of CD69 compared to the other groups (Figure 5A).

In contrast to the low prevalence of NK cells in the tumors, a significant increase in NK number and activation status, as determined by CD69, NKG2D, and intracellular IFN-γ, was detected in the TDLNs of the VSV-NDV-treated tumors on day 2 post-treatment (Figure 5B). In addition, an increase in NK cell maturation (CD11b^+^/CD27^−^) and activation, as evidenced by intracellular IFN-γ and NKG2D expression on NK cells, was observed in the spleen on day 2 after VSV-NDV treatment compared to PBS (Figure 5B). Furthermore, an increase in DC numbers in the TDLNs of the VSV-NDV treated tumors was similarly observed, as well as a corresponding increase in CD86 and MHC-I expression (Figure 5C), indicating that these cells are activated.

### 3.5. The In Vivo Depletion of CD8^+^ T Cells Reverses the Beneficial Effect of rVSV-NDV Treatment in the B16 OVA

**Figure 6:**
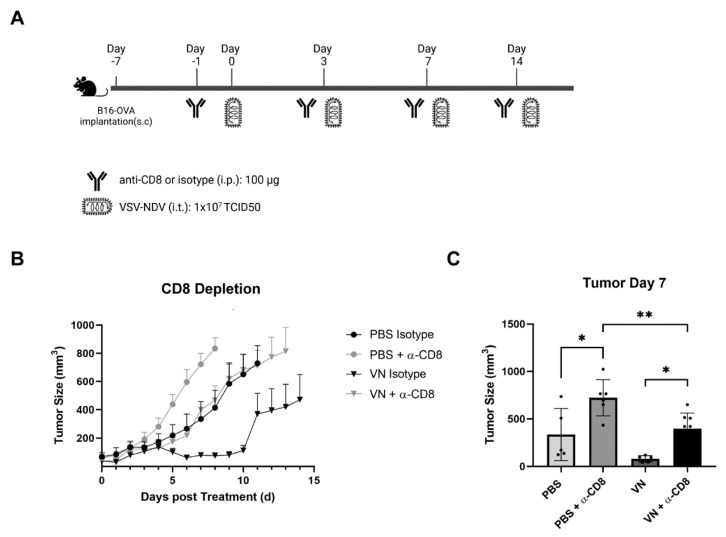
In vivo CD8^+^ T cell depletion abrogates the therapeutic effects of rVSV-NDV treatment. (**A**) The experimental set-up of CD8^+^ T cell depletion in B16OVA tumor-bearing mice treated with oncolytic rVSV-NDV vectors in vivo. Mice (N = 5–6) received either CD8 depletion antibody or IgG isotype control in 100 µL PBS by intraperitoneal injection 6 days after tumor implantation. One day later, complete depletion was confirmed by flow cytometry, and mice received VSV-NDV (1 × 10^7^ TCID50) or PBS in a 50 µL volume by intratumoral injection on day 0, 3, 6, and 14. (**B**) Tumor growth was monitored daily. (**C**) Tumor weights on day 7 post-treatment start. Data points indicate values of individual replicates (and group means ± SD are presented as bars); * indicates *p* < 0.05 and ** indicates *p* < 0.01.

In order to investigate the role of CD8^+^ T cells in the therapeutic effect of the virus, CD8^+^ T cells were transiently depleted using systemically applied antibodies after the engraftment of B16OVA tumors (Figure 6A). As expected based on previous results, treatment with VSV-NDV in the antibody isotype control group led to excellent tumor control compared to PBS treatment. Although variation in responses led to an increase in mean tumor volume after 10 days, tumor volumes stabilized again and remained controlled in comparison to the PBS treatment groups. rVSV-NDV treatment in the context of CD8 depletion led to tumor growth kinetics that were indistinguishable from the PBS + isotype treatment group, while PBS treatment in the CD8-depletion group led to even further enhanced tumor growth (Figure 6B). A comparison of individual tumor volumes on day 7 further highlighted the complete abrogation of the therapeutic effects of rVSV-NDV treatment by CD8^+^ T cell depletion (Figure 6C). Together, these data strongly indicate that the therapeutic effects of rVSV-NDV are highly CD8^+^ T cell-dependent.

### 3.6. Virus Infection of Cancer Cells Leads to Activation of Human T Cells and NK Cells

**Figure 7:**
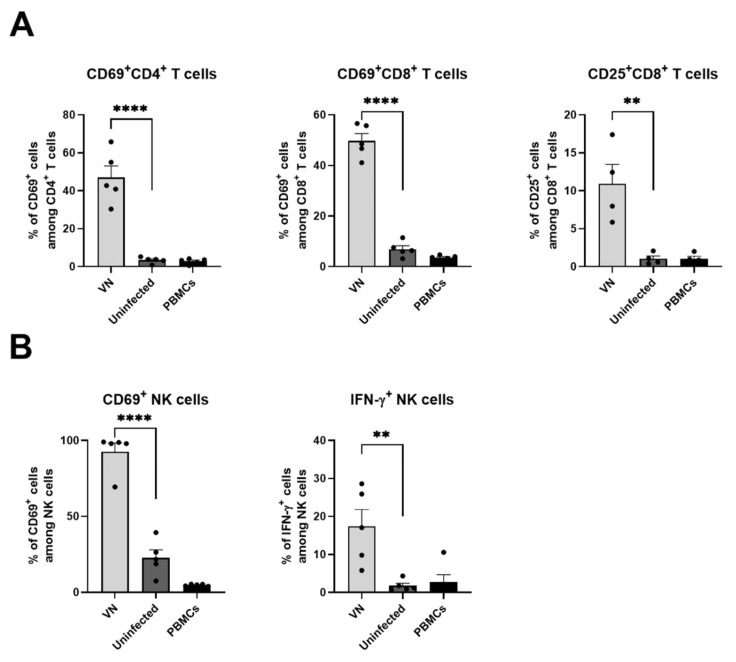
Human T cells and NK cells are activated by VSV-NDV-infected cancer cells in vitro. HepG2 cells pre-infected with VSV-NDV were co-cultured with human PBMCs in a 1:1 ratio for 24 h before analysis by flow cytometry. PBMCs that were not co-cultured served as controls. Activation was measured by (**A**) expression of CD69 on CD4^+^ and CD8^+^ T cells and CD25 expression on CD8^+^ T cells or (**B**) expression of CD69 and IFN-γ on NK cells (N = 4–5 individual experiments); ** indicates *p* < 0.01 and **** indicates *p* < 0.0001.

In order to predict whether the immune cell responses observed in the murine model would translate to the human system, we established an in vitro co-culture system of tumor cells infected with VSV-NDV and immune cells. Human hepatocellular carcinoma (HepG2) cells were pre-infected with VSV-NDV and combined with human PBMCs, followed by flow cytometry analysis 24 h later. Interestingly, the co-culture of PBMCs with VSV-NDV-infected cancer cells mediated a significant upregulation of CD69 levels on human CD4^+^ and CD8^+^ T cells, while CD25 was additionally upregulated on CD8^+^ T cells (Figure 7A). Human NK cells co-cultured with VSV-NDV-infected tumor cells demonstrated an increase in CD69 and IFN-γ expression (Figure 7B). Taken together, these results indicate a potential immune-stimulatory activity of VSV-NDV in the human system.

## 4. Discussion

Despite the widespread adoption of immune checkpoint inhibitors (ICI) as the standard of care across various cancer types, such as unresectable or metastatic melanoma, metastatic non-small cell lung cancer (NSCLC), bladder cancer, Hodgkin lymphoma, advanced renal cell carcinoma (RCC), or hepatocellular carcinoma (HCC), a substantial proportion of patients fail to respond due to the prevailing immune-suppressive TME [17], [18,19]. The transition of the immunologically “cold” TME into a “hot” state represents a fundamental objective in current cancer therapy. Strategies aiming at reprogramming the TME include emerging approaches like cytokine therapy, cancer vaccines, and oncolytic viruses [20]. In the work presented here, we demonstrate that the therapeutic effects of oncolytic VSV-NDV correspond with intricate modulations of immune cell subsets across distinct anatomical compartments. Notably, treatment with VSV-NDV instigated the robust activation and expansion of tumor antigen-specific and effector T cell subsets, which were not confined to the local tumor site, but were also evident systemically, implicating a broader immune activation and highlighting its potential therapeutic utility in metastatic settings.

Notably, when CD8^+^ T cells were depleted prior to VSV-NDV treatment in the B16OVA model, the beneficial effects of the virus were abolished, highlighting a central role of this cell type in the mechanism of action of this OV therapy. CXCL10, a chemokine known for its role in immune cell trafficking, and shown to be upregulated in the tumor following VSV-NDV treatment (Figure 1C), has significant beneficial effects in the TME, particularly in enhancing CD8^+^ T cell responses [21,22]. We suggest that CXCL10 attracts CD8^+^ T cells into the tumor, where they generate robust anti-tumor effects, although the enhanced T cell infiltration in the distant (uninfected) tumor would indicate the presence of an additional, broader immune mechanism. Furthermore, TCR-independent activation of human T cells and NK cells was observed in an in vitro co-culture set up with pre-infected tumor cells, suggesting a multimechanistic immune-stimulatory potential of oncolytic VSV-NDV that could translate to a clinical setting in humans.

These findings are consistent with those observed from other oncolytic viruses. In B16-CD20 melanoma, treatment with oncolytic measles virus led to an increase in CD8^+^ T cells in the tumor [23]. Similarly, treatment with VSV in the B16OVA model led to an increase in CD8+ tumor-infiltrating lymphocytes (TILs) [24]. VSV-GP, a chimeric variant of VSV, elicited an increase in CD4^+^ and CD8^+^ T cells within treated B16OVA tumors, albeit generating a weak OVA-specific CD8^+^ T cell response [25]. Previous studies have reported an even weaker antigen-specific immune response for VSV wildtype, attributed to the direct infection and destruction of tumor-associated DCs [26]. Other oncolytic viruses, such as Zika virus, vaccinia virus, and adenovirus, have similarly been reported to induce cytotoxic T cell infiltration and activation in the tumor microenvironment [27,28,29].

A central hypothesis of our research is that fusogenic oncolytic virus vectors mediate enhanced immune-stimulatory effects through their heightened immunogenicity. In a breast cancer model, a recombinant fusogenic VSV variant carrying reovirus fusion-associated small transmembrane (FAST) proteins and the VSV(MΔ51) backbone (a more immunogenic variant of VSV) induced a significant increase in iNKT cells, NK cells, CD8^+^ and CD4^+^ T cells, and DCs in the tumor, compared to untreated and VSV-GFP-treated tumors [30], which is consistent with our observations of immune cell responses to VSV-NDV treatment. This is likely due to the potent induction of ICD by the virus-mediated cell fusion [14,31].

A valid concern in the OV field is that viral vectors may induce strong anti-viral immune responses, which could counteract the therapy and detract from an efficient induction of anti-tumoral immunity. It was recently reported that oncolytic VSV-IFN-β led to the expansion of dominant antiviral effector CD8^+^ T cells, which coincided with the timing of an observed reduction in anti-tumor T cell populations [32]. Interestingly, in the model used here, we demonstrated a strong correlation between the presence of anti-viral and tumor-specific CD8^+^ TILs, suggesting that VSV-NDV therapy not only does not distract from the induction of tumor-specific immunity, but it might even promote it. Notably, the activation of antiviral memory T cells has previously been associated with tumor growth inhibition, and viral peptides have been shown to sensitize B16 melanoma to PD-L1 blockade [30]. Furthermore, prior immunization with NDV has been linked to superior tumor clearance and survival in mice [31], underscoring the potential of antiviral immune responses to mediate potent anti-tumor effects, which seems to also hold true for VSV-NDV.

This study additionally revealed an early increase in innate immune cells, marked by the elevation of activated iNKT cells, NK cells, and DCs in TDLNs from the treated tumor, implicating their involvement in the initial immune response. The collective findings from numerous studies indicate that NK cells frequently impact favorably on the therapeutic outcomes of OV therapy [33,34,35,36,37,38]. This phenomenon can be explained by the pivotal role of the interaction between NK cells, T cells, and DCs. NK cells and T cells have the capability to recruit classical type 1 DCs (cDC1s) through the secretion of chemokines [39], and multiple reports have documented the occurrence of the cross-priming of CD8^+^ T cells by DCs in response to virotherapy [40,41,42,43]. It should be noted, however, that NK cells also play an important role in clearing oncolytic viruses, often limiting their oncolytic efficacy [44]. Therefore, the dual role of NK cells, driving both antitumor responses as well as antiviral responses, must be carefully considered. Although a stronger innate immune cell response was expected within the virus-treated TME, our findings may be specific to the subcutaneous tumor setting of the applied model or to the timepoints investigated.

While it was beyond the scope of this work to characterize the precise mechanism and cross-talk among the individual immune cell types, a critical dependence on the cytotoxic T cell response for the therapeutic outcome of VSV-NDV therapy is strongly implicated by the complete ablation of tumor responses in the context of CD8^+^ T cell depletion. While the importance of CD8^+^ T cells in OV therapy is not completely unexpected, our results imply that the cytotoxic T cell response may even represent the more potent mechanism of action compared to the direct oncolytic effect. The fusion-mediated tumor lysis seems to be a critical driver of these T cell responses, as a concerted consequence of the therapeutic reprogramming of the TME that promotes immune cell infiltration and activation.

While these data provide a first proof-of-concept of the dynamic immune cell responses to this novel OV therapy, it is important to recognize the limitations of the B16OVA model. The artificial OVA antigen leads to potent immune responses that likely exceed those against natural TAAs, potentially leading to an overestimation of efficacy [45,46,47,48]. This model served as a preliminary screening tool to identify immune cell responses to VSV-NDV therapy, which will certainly need to be validated in more immunologically predictive models and compared across distinct tumor indications.

## 5. Conclusions

This work unveiled an orchestrated activation of various immune cell populations, including cytotoxic T cells, iNKT cells, DCs, and NK cells, reflecting a multifaceted global immune response which likely contributes to the potent anti-tumor effects of VSV-NDV. These findings highlight the intricate and dynamic interplay between local and systemic immune responses and provide a potential mechanism for the synergistic effects of VSV-NDV in combination with immune checkpoint inhibitors [16] and adoptive T cell therapy that were previously observed [15]. Having characterized substantial immune-modulatory effects in the TME and lymphoid tissues following oncolytic VSV-NDV therapy, the rationale for combining this therapy with other immunotherapies is strongly supported. Moving forward, VSV-NDV represents a promising OV-based immunotherapy platform for solid cancers.

## 6. Patents

J.A. holds a patent for the development and use of VSV-NDV as an oncolytic therapy of cancer.

## Figures and Tables

**Table 1:** Primer sequences.

Target	Forward (5′-3′)	Reverse (5′-3′)
GAPDH	GCCTTCTCCATGGTGGTGAA	GCACAGTCAAGGCCGAGAAT
IFN-γ	TCAAGTGGCATAGATGTGGAAGAA	TGGCTCTGCAGGATTTTCATG
IL-15	CATCCATCTCGTGCTACTTGTGTT	TGGCTCTGCAGGATTTTCATG
T-bet	TAAGCAAGGACGGCGAATGTT	TGCCTTCTGCCTTTCCACAC
IFN-α	ATGGCTAGRCTCTGTGCTTTCCT	GTCTCGTCTTYAGACCTCTCGGGA
IFN-β	GGAGATGACGGAGAAGATGC	CCCAGTGCTGGAGAAATTGT
CXCL10	CCA AGT GCT GCC GTC ATT TTC	GGC TCG CAG GGA TGA TTT CAA

## Data Availability

The data are available within this article. Original raw data are available upon request to the corresponding author.
